# Number line estimation strategies in children with mathematical learning difficulties measured by eye tracking

**DOI:** 10.1007/s00426-015-0736-z

**Published:** 2015-12-26

**Authors:** Jaccoline E. van’t Noordende, Anne H. van Hoogmoed, Willemijn D. Schot, Evelyn H. Kroesbergen

**Affiliations:** Department of Special Education: Cognitive and Motor Disabilities, Utrecht University, P.O. Box 80140, 3508 TC Utrecht, The Netherlands

## Abstract

**Introduction:**

Number line estimation is one of the skills related to mathematical performance. Previous research has shown that eye tracking can be used to identify differences in the estimation strategies children with dyscalculia and children with typical mathematical development use on number line estimation tasks. The current study extends these findings to a larger group of children with mathematical learning disabilities (MLD).

**Method:**

A group of 9–11-year-old children with MLD (*N* = 14) was compared to a control group of children without math difficulties (*N* = 14). Number line estimation was measured using a 0–100 and a 0–1000 number-to-position task. A Tobii T60 eye tracker was used to measure the children’s eye movements during task performance.

**Results:**

The behavioral data showed that the children with MLD had higher error scores on both number lines than the children in the control group. The eye tracking data showed that the groups also differed in their estimation strategies. The children with MLD showed less adaptation of their estimation strategies to the number to be estimated.

**Conclusion:**

This study shows that children with MLD attend to different features of the number line than children without math difficulties. Children with math difficulties are less capable of adapting their estimation strategies to the numbers to be estimated and of effectively using reference points on the number line.

**Electronic supplementary material:**

The online version of this article (doi:10.1007/s00426-015-0736-z) contains supplementary material, which is available to authorized users.

## Introduction

Children with mathematical learning difficulties (MLD) have problems in estimating the positions of numbers on a number line; their estimations deviate more from the requested number as in typically developing children (e.g. Geary, Hoard, Byrd-Craven, Nugent, & Numtee, 2007; Geary, Hoard, Nugent, & Byrd-Craven, 2008; Van’t Noordende & Kolkman, 2013). However, the underlying causes of their estimation difficulties remain unclear. It is possible that they have problems in using estimation strategies, as the use of estimation strategies is related to number line performance (Ashcraft & Moore, 2012; Newman & Berger, 1984). In the last decade, there has been an increasing interest in the use of eye tracking to measure number processing (Hartmann, 2015; Mock, Huber, Klein, & Moeller, this issue). The aim of the current study is to unravel possible differences in number line estimation strategies between children with MLD and children with typical mathematical development using eye tracking.

Most of the previous research on number line estimation assumed that the estimations reflect an internal, mental representation of a number line. Participants’ estimations are modeled along a linear or logarithmic regression line, which leads to the assumption that magnitudes are represented logarithmic or linearly (e.g. Booth & Siegler, 2006; Siegler & Booth, 2004). Recently, researchers have criticized this assumption and developed new models like the two-linear model (Ebersbach, Luwel, Frick, Onghena, & Verschaffel, 2008; Moeller, Pixner, Kaufmann, & Nuerk, 2009) and the proportional judgment models (e.g. Barth & Paladino, 2011). These models are based on the view that children’s actual scores on an estimation task are influenced by the strategies they use and therefore do not allow for direct inferences on their mental representation. As such, a logarithmic estimation pattern does not necessarily imply an underlying logarithmic magnitude representation, but could also be caused by the inability to use an adequate estimation strategy (Sullivan & Barner, 2014). Thus, more research is needed to give insights into the actual strategies that children use during an estimation task.

The proportional judgment models propose the use of reference points (beginning, mid, and end) to estimate the target number on a line. These models have been tested with cyclic power models. It has been found that cyclic power models fit number line estimations better than linear and logarithmic models (Barth & Paladino, 2011; Friso-van den Bos et al., 2015; Huber, Moeller, & Nuerk, 2014; Rouder & Geary, 2014; Slusser, Santiago, & Barth, 2013), suggesting reference points are indeed used for number line estimation. This has been confirmed by eye tracking studies (Schneider et al., 2008; Sullivan, Juhasz, Slattery, & Barth, 2011). These studies examined at which aspects of the number line people fixate, and thus attend to before giving a response. It was found that the amount of fixations peaks around the beginning, mid- and endpoints of the line both in adults (Sullivan et al., 2011) and in children (Schneider et al., 2008), indicating that these points are used as reference points.

Developmental trends in number line estimation strategy use have been found during the first years of primary school. The use of the beginning, mid-, and endpoints seems to gradually develop from grade 1 onwards, starting with the use of the beginning point, than the beginning and endpoints and finally the use of all three points (Ashcraft & Moore, 2012; Friso-van den Bos et al., 2015; Rouder & Geary, 2014; Schneider et al., 2008; White & Szűcs, 2012). The use of all three reference points seems to appear earlier in development and seems to be more stable for smaller number ranges than for larger number ranges (Ashcraft & Moore, 2012). Moreover, Newman and Berger (1984) found that younger children mainly reported using the beginning point of the line, whereas grade 3 children used the reference points more flexibly, according to their self-reports. The older children adapted their estimation strategy to the specific number to be estimated, i.e. they used the reference point closest to the target number.

To summarize, the described studies indicate that people make use of reference points when estimating numbers on a number line and an increasing use of different reference points becomes visible with increasing age and numerical experience. These estimation strategies are related to performance on number line tasks. For example, Newman and Berger (1984) found that children who report using the reference points on the number line adaptively are more accurate in their estimations than children with a less flexible strategy use. Likewise, Sullivan and Barner (2014) suggest that children who have problems with proportional reasoning will score low on a number line estimation task, because of problems with using adequate estimation strategies. This implies that the seemingly less linear—or more logarithmic—number line estimation patterns of children with MLD (e.g. Geary et al., 2007, 2008) could actually be the reflection of inabilities to make use of efficient estimation strategies. This would be in line with other domains of mathematics, in which children with MLD also have shown to display difficulties in strategy use (Torbeyns, Verschaffel, & Ghesquière, 2004, 2006). They are likely to experience such problems on number line tasks as well, because they lag behind in mathematical skills needed to use estimation strategies. For example, the use of reference points on the number line is related to arithmetic procedures (Link, Nuerk, & Moeller, 2014) and children need to be aware that the midpoint of the line corresponds to the midpoint of the number range to correctly use it as a reference point (Ashcraft & Moore, 2012; White & Szűcs, 2012). This suggests that children who lag behind in mathematical abilities should have problems using reference points on number line tasks. Moreover, children with MLD often have difficulties in spatial cognition (Swanson & Jerman, 2006), a skill that is also needed to make use of proportional estimation strategies.

A recent study indeed showed differences in estimation strategies between children with MLD and a control group without MLD. As expected, children with MLD made less use of reference points compared to children without MLD. Surprisingly, however, the children with MLD looked *more* at the midpoint than the control group (Van’t Noordende & Kolkman, 2013). Van’t Noordende and Kolkman (2013) suggested that children with MLD know they can use the midpoint as a reference point, but do not adapt their estimation strategy to the number that has to be estimated. This hypothesis could not be confirmed, since strategy use was examined on task level (measuring strategy use across all estimated numbers) instead of item level (measuring strategy use per estimated number). Two other studies did assess differences in functionality of strategy use between children with and without developmental dyscalculia (Schot, Van Viersen, Van’t Noordende, Slot, & Kroesbergen, 2015; Van Viersen, Slot, Kroesbergen, Van’t Noordende, & Leseman, 2013). They defined the functionality of an estimation strategy by the proximity of the reference point to the number that had to be estimated, for example using the beginning point on a 0–100 number line to estimate the number 18. A dysfunctional estimation strategy was defined as using a reference point far away from the number that had to be estimated, for example using the endpoint on a 0–100 number line to estimate the number 18. A case study on a 9-year-old girl with developmental dyscalculia showed that the reference point used by this girl was dysfunctional in 26 % of the trials, whereas only 8 % of the estimation strategies used by the control group was dysfunctional (Van Viersen et al., 2013). Schot et al. (2015) included two children with developmental dyscalculia and plotted fixation patterns on the number line with respect to both the numbers that had to be estimated and the number that was estimated by the children. They found that the fixations of the children with developmental dyscalculia were more scattered across the number line and farther away from both the target number and the response than in the control group, indicating no—or at least a weaker—relation between the target number or the response and looking behavior. Together, these results suggest that children with MLD have problems in using functional estimation strategies in number line estimation. However, these studies were case studies and did not statistically test differences in functionality of strategy use. Therefore, in the current study strategy use on number lines 0–100 and 0–1000 will be tested with eye tracking in a larger group of children with MLD. The goal is to assess whether children with MLD differ in strategy use from children without MLD and more specifically, whether children with MLD use less functional estimation strategies than children without MLD. This will help us to understand the specific difficulties of children with MLD on number line estimation.

## Method

### Participants

A group of 14 children (2 boys and 12 girls; *M* age = 11.09, SD = 1.10 years) with mathematical learning difficulties (MLD) participated in this study.^1^ These children were recruited via the ambulatory service of Utrecht University specialized in dyscalculia to which they were referred because of problems with mathematical learning in school. All children in the specified age range of 10–12 years and whose parents gave permission to use test results for research purposes were included in the study. On average they lag behind 19 months in automatization in mathematics compared to typically developing children. All children met the criteria for dyscalculia used in the centre: they scored below the 10th percentile on standardized math tests [both a timed test with basic facts (TempoToets Automatiseren) and a standard national criterion-based math test (CITO) that is administered twice a year in almost every classroom in the Netherlands]. The CITO mathematics test consists of grade-appropriate mathematics problems, primarily word problems that cover a wide range of mathematics domains such as measurement, time, and proportions. Scores are converted into five categories: 0–10, 10–25, 25–50, 50–75, and 75–100 %. All MLD children scored in the lowest category on at least two assessments.

The age-matched control group consisted of 14 children (3 boys and 11 girls; *M* age = 10.71, SD = 0.89 years) without MLD, selected from primary schools. Their teachers did not report any known mathematical difficulties and all children scored at or above mean level on the CITO mathematics test (6 children scored between 75 and 100 %, 5 children scored between 50–75 % and 3 children scored between 25 and 50 %).

### Procedure

All children were tested on a computer with Tobii T60 eye tracker in the Pedagogics lab at Utrecht University. The temporal resolution of the Tobii T60 is 60 Hz. The spatial resolution is 0.2°. A nine-point calibration was used. For all children, the 0–100 number line was administered first and the 0–1000 number line second.

### Instruments

Two number line tasks were used to measure number line estimation: (1) a 0–100 number line task, and (2) a 0–1000 number line task. An empty number line was presented on the computer screen with numbers only at the beginning and endpoints (i.e. 0 and 100, or 0 and 1000, respectively). Then the number that had to be estimated was presented beneath the number line. Children were asked to read the number aloud and then estimate its position on the number line by placing the mouse cursor on the line. To make sure the numbers that had to be estimated were more or less equally distributed over the number line, the number line was divided into 33 equal sections and one number from each section was randomly selected to be used in the task. For the 0–100 number line task, the used numbers were: 3, 5, 9, 10, 14, 18, 19, 24, 27, 28, 32, 34, 37, 41, 43, 46, 49, 53, 57, 60, 61, 64, 66, 72, 74, 78, 80, 83, 87, 89, 91, 96, 99; for the 0–1000 number line task, the used numbers were: 4, 36, 68, 104, 135, 153, 201, 230, 261, 277, 308, 354, 385, 398, 422, 469, 510, 528, 542, 594, 613, 636, 684, 697, 723, 763, 804, 844, 862, 880, 919, 958, 996. The same numbers were presented to each child but in a different random order.

### Data analysis

#### Behavioral performance

To quantify performance on the number line, the absolute error was calculated and expressed as a proportion of the range of the number line (called the percentage absolute error) using the following formula: (response − target number)/range number line (100 or 1000) × 100 (Siegler & Booth, 2004). Furthermore, for each participant the conventional linear and logarithmic model fit was computed by conducting a regression with the estimated number (response) as dependent variable and the target number (equaling the correct answer) as independent variable. Finally, a two-cycle power model was fitted to the individual data as an index of beginning point, midpoint and endpoint use (Slusser et al., 2013). For all models, *R*^2^ was used as an index of model fit. A MANOVA was used to test for possible group differences in absolute error and model fit on each number line.

#### Eye movements

Eye movements were analyzed using Matlab (Mathworks Inc). They were classified as fixations when the absolute speed of the eyes was lower than 3 m/s for at least three consecutive samples (50 ms). Fixations were pooled if they were within 0.5 cm^2^ of each other. Only fixations that fell within 3.5 cm above or below the number line and occurred between the start of the stimulus presentations and the participants’ response were included in the analyses. The number that had to be estimated was presented more than 3.5 cm under the number line, and fixations on this number were thus excluded from the analyses (Schot et al., 2015).

#### Estimation strategies

To gain insights into the estimation strategies that the children used, we assessed whether children made use of the reference points (beginning point, midpoint, or endpoint). Fixations within a margin of 5 % from the beginning, mid-, or endpoint were classified as fixations on these respective reference points. When the fixations on a particular trial were confined to just one of these reference points, the estimation strategy for this trial was classified as a beginning, mid-, or endpoint estimation strategy depending on the reference point the child used. When there were fixations on multiple references the estimation strategy was classified as such (i.e. begin and mid, begin and end, mid and end, or all references). When there were no fixations on the references, but all fixations were within 5 % of the correct answer, and the given answer was within 5 % around the correct answer, the estimation strategy was classified as automatized. In trials with no fixations on the references and no fixations around the correct answer (within 5 %), the estimation strategy was classified as guess when all fixations were within 10 % of the given answer and classified as no references (NoRefs) when fixations were scattered over the number line. Trials in which no eye movement data were available (for example due to movement of the child) were excluded from the analyses. In total, 1.84 % of the trials was lost due to this constraint. We calculated the percentage of trials in which the children used each of the estimation strategies in both tasks. A MANOVA was used to test for possible group differences in strategy use on each number line. To avoid problems with dependency because the strategies sum up to 100 %, the ‘no references’ category was not included in the MANOVA.

#### Functionality of fixations

To examine the functionality of the eye-fixation behavior, the horizontal position of the fixations was plotted against the target number and against the response for each participant separately. The number line was then divided into three equal segments (0–33/0–333, 34–66/334–666 and 67–100/667–1000) to examine whether the fixations were near or farther away from the target number and the response over trials. For each trial, the percentage of fixations that fell in the same segment as the target number (near), in the segment next to the correct segment (in between), and two segments away^2^ from the correct segment (far) was calculated per participant separately for each task. The same percentages were calculated relative to the participants’ response. For the control group, the mean of these percentages was calculated and plotted. Because of the large variance in the MLD group, results were plotted for each participant separately.

#### Adaptive strategy use

Finally, to assess whether strategy use was adaptive (i.e. related to the number that had to be estimated), we calculated the percentages of the estimation strategies used for both the MLD group and the control group for each trial separately. Based on the pattern of estimation strategy uses across the number range in the control group (see Online Resource 1), the number lines were then again divided into three equal sections (0–33, 34–66, 67–100; 0–333, 334–666, 667–1000) and the percentages of the use of beginning point, midpoint, and endpoint used within each section were compared between the MLD group and the control group.

##### Use of estimation strategies per number section

A repeated measures MANOVA was used with group (MLD, control) as independent variable, estimation strategy (beginning point, midpoint, and endpoint) as within subjects factor, and section of the number line as measure, to examine if there were differences between the MLD and control group in which estimation strategy they used most in each number section. Whenever the assumption of sphericity was violated, the Greenhouse-Geisser correction coefficient ($\hat{\varepsilon }$) is reported together with the uncorrected degrees of freedom and the corrected *p* value.

##### Use of most adaptive strategy per number section

A MANOVA was used to test for differences between the groups in use of the (theoretically) most adaptive strategy for each number section, with group as independent variable and the use of beginning point in the low number section, the use of midpoint in the medium number section and the use of endpoint in the high number section as dependent variables (since these are theoretically the most adaptive strategy for each number section).

For statistical analysis, *α* = .05 was used. Effect sizes were classified according to the criteria of Cohen (1988): *η*^2^ ≥ .01 is small, *η*^2^ ≥ .06 is medium, *η*^2^ ≥ .13 is large.

## Results

### Behavioral performance

The descriptive statistics of the behavioral outcomes are displayed in Table 1. The results of the MANOVA showed a large multivariate effect on both the number line 0–100, Wilks’ Lambda = 0.62, *F* (4, 23) = 3.57, *p* = .021, *η*^2^ = .38, and the number line 0–1000, Wilks’ Lambda = 0.65, *F* (4, 23) = 3.04, *p* = .038, *η*^2^ = .35. Univariate effects are shown in Table 1. On both number line tasks, the linear fit was higher in the control group than in the MLD group and the percentage absolute error was lower in the control group than in the MLD group. The two-cycle power fit was also higher in the control group than in the MLD group, although this difference was only marginally significant on the number line 0–100. There was no difference between the groups in logarithmic fit, although the effect size on the number line 0–100 was medium to large.

**Table 1:** Descriptive statistics and univariate between-group effects of behavioral performance on the number line 0–100 and 0–1000

	MLD		Control		Univariate effects				
	*M*	SD	*M*	SD	*F*	*df1*	*df2*	*p*	*η* ^*2*^
Number line 0–100									
Percentage absolute error	6.66	2.58	4.14	1.44	10.14	1	26	.004	.28
Linear *R* ^*2*^	.93	.05	.97	.02	9.98	1	26	.004	.28
Logarithmic *R* ^*2*^	.76	.05	.79	.03	3.59	1	26	.069	.12
Two-cycle *R* ^*2*^	.90	.12	.96	.03	4.16	1	26	.052	.14
Number line 0–1000									
Percentage absolute error	10.86	5.44	5.39	1.57	13.06	1	26	.001	.33
Linear *R* ^*2*^	.79	.20	.96	.02	9.40	1	26	.005	.27
Logarithmic *R* ^*2*^	.60	.10	.63	.04	0.85	1	26	.365	.03
Two-cycle *R* ^*2*^	.63	.37	.94	.03	9.78	1	26	.004	.27

*N*_MLD group_ = 14; *N*_control group_ = 14

### Estimation strategies

The percentage of the use of each strategy on both tasks is reported in Table 2. There were no large differences in strategy use on the number line 0–100 between the groups. The MANOVA showed a large—although non-significant—effect, Wilks’ Lambda = 0.61, *F* (9, 18) = 3.57, *p* = .317, *η*^2^ = .39. Univariate results are reported in Table 2. There was a medium—although non-significant—difference in use of the beginning point, reflecting more use of this estimation strategy in the control group as compared to the MLD group. Besides this, the MLD group showed a higher percentage of guess than the control group. This effect was non-significant but nevertheless had a medium effect size.

**Table 2:** Descriptive statistics and univariate between-group effects of estimation strategies on the number line 0–100 and 0–1000

	MLD		Control		Univariate effects				
	*M*	SD	*M*	SD	*F*	*df1*	*df2*	*p*	*η* ^*2*^
Number line 0–100									
Beginning point	6.06	7.13	10.39	8.87	2.03	1	26	.167	.07
Midpoint	26.62	12.59	27.06	9.32	0.01	1	26	.918	.00
Endpoint	9.74	7.44	12.77	7.15	1.21	1	26	.282	.04
Begin-mid	3.25	4.36	2.38	2.70	0.40	1	26	.533	.02
Begin-end	1.95	2.26	1.08	1.92	1.20	1	26	.284	.04
Mid-end	6.93	6.22	4.76	3.08	1.36	1	26	.254	.05
All refs	1.52	3.52	0.87	1.85	0.37	1	26	.546	.01
Automatized	6.71	6.31	5.84	4.82	0.17	1	26	.687	.01
Guess	5.41	7.34	2.16	2.20	2.51	1	26	.125	.09
Number line 0–1000									
Beginning point	4.11	2.55	12.55	8.14	13.72	1	26	.001	.35
Midpoint	28.79	9.26	26.62	11.96	0.29	1	26	.597	.01
Endpoint	8.23	5.24	9.09	4.75	0.21	1	26	.651	.01
Begin-mid	1.95	2.55	3.68	4.15	1.77	1	26	.195	.06
Begin-end	0.87	1.42	1.95	2.55	1.92	1	26	.177	.07
Mid-end	6.71	5.21	3.90	2.50	3.32	1	26	.080	.11
All refs	2.60	3.54	0.65	1.29	3.75	1	26	.064	.13
Automatized	5.63	6.38	7.58	7.59	0.54	1	26	.469	.02
Guess	8.44	10.58	2.60	4.26	3.68	1	26	.066	.12

*N*_MLD group_ = 14; *N*_control group_ = 14

The MANOVA on the number line 0–1000 showed a large group effect, Wilks’ Lambda = 0.41, *F* (9, 18) = 2.93, *p* = .025, *η*^2^ = .59. There was a large and highly significant difference between the groups on use of the beginning point, which was less frequently used in the MLD group as compared to the control group. Furthermore, there were marginally significant effects for the midpoint-endpoint estimation strategy, the all reference points’ estimation strategy and for guessing. The MLD group made slightly more use of these strategies as compared to the control group.

### Functionality of fixations

**Fig. 1:**
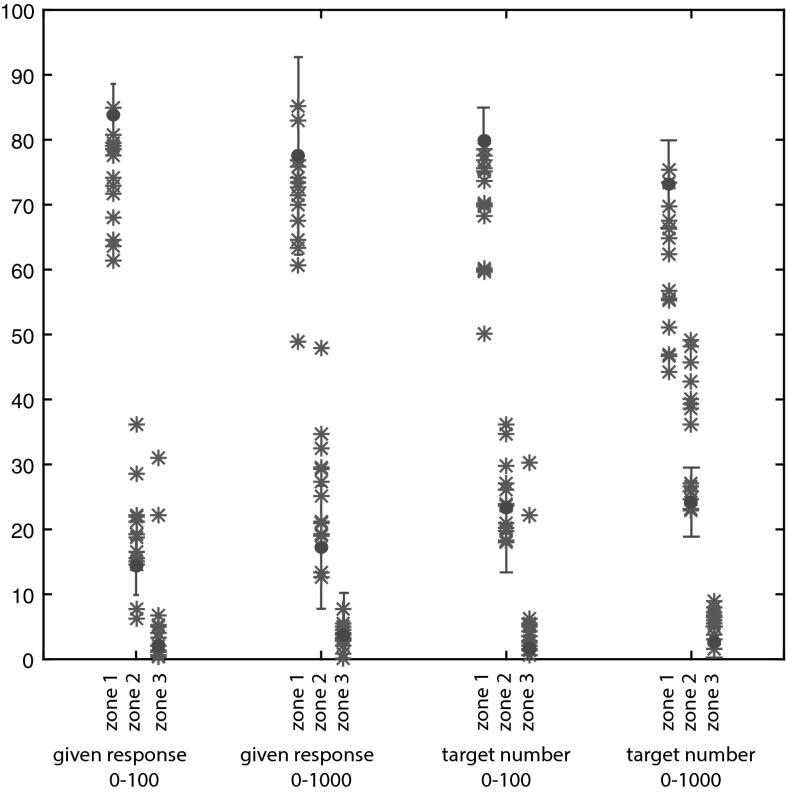
Percentages fixations per zone (*1* near, *2* in between, *3* far) in relation to the target number (correct answer) and response on the number line 0–100 and the number line 0–1000. The *error bars* represent the mean of the control group (±1 SD). The *stars* represent the individual participants from the MLD group

To gain insights into the functionality of the fixations, the individual fixation patterns were plotted in relation to the target number and the response (see Online Resource 2). The percentage fixations being near (in the same zone), far (two zones away), or in between near and far (one zone away) from the target/estimated number are displayed in Fig. 1. It was found that the fixations of the children in the MLD group were farther away from the target number. This difference was not solely caused by the fact that the responses of the children in the MLD group were also farther away from the target number, since the fixations of the children in the MLD group were also farther away from their own responses.

### Adaptive strategy use

**Fig. 2:**
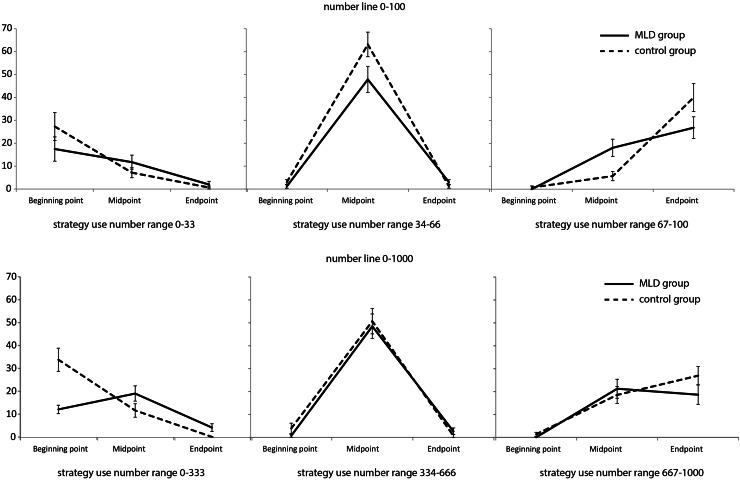
Mean percentage strategy use per number section on the number line 0–100 and the number line 0–1000

Finally, the adaptive use of estimation strategies was examined. The descriptive statistics of strategy use per number section and per group are displayed in Fig. 2.

#### Use of estimation strategies per number section

The repeated measures MANOVA for the 0–100 task showed a significant interaction between group and strategy use, Wilks’ Lambda = 0.59, *F* (6, 100) = 5.11, *p* < .001, *η*^2^ = .24. The assumption of sphericity was violated for all three number sections, so degrees of freedom for univariate testing have to be corrected by $\hat{\varepsilon }$. Univariate testing showed a medium—although non-significant—difference on strategy use between the groups on the 0–33 section, *F* (2, 52) = 1.90, $\hat{\varepsilon }$  = .637, *p* = .176, *η*^2^ = .07. Pairwise comparisons showed that the MLD group used the beginning point strategy (*p* = .017) and the midpoint strategy (*p* = .012) significantly more than the endpoint strategy. There was no difference between the use of beginning point and midpoint strategy (*p* = .414). The control group used the beginning point strategy more than the midpoint (*p* = .011) and endpoint strategy (*p* = .001). They also made more use of the midpoint strategy than the endpoint strategy (*p* = .012).

On the 34–66 section there was also a marginally significant, medium sized univariate interaction effect between strategy use and group, *F* (2, 52) = 3.54, $\hat{\varepsilon }$  = .556, *p* = .066, *η*^2^ = .12. The MLD group group used the midpoint strategy more than the beginning point (*p* < .001) and endpoint strategy (*p* < .001). There was no difference between the use of beginning point and endpoint strategy (*p* = .272). Similar results were found for the control group on this number section. They used the midpoint strategy more than the beginning point (*p* < .001) and endpoint strategy (*p* < .001) and there was no difference between the use of beginning point and endpoint strategy (*p* = .272).

The univariate interaction effect between strategy use and group was significant on the 67–100 section, *F* (2, 52) = 5.85, $\hat{\varepsilon }$  = .655, *p* = .014, *η*^2^ = .18. The MLD group group used the endpoint strategy (*p* < .001) and the midpoint strategy (*p* < .001) more than the beginning point strategy. There was no difference between the use of midpoint and endpoint strategy (*p* = .171). For the control group, all pairwise comparisons on this number section were significant. The control group used the endpoint strategy more than the midpoint (*p* < .001) and beginning point strategy (*p* < .001) and the midpoint strategy more than the beginning point strategy (*p* = .047).

To summarize, the results on the number line 0–100 indicated that the MLD group used the midpoint strategy equally often as the beginning point/endpoint in, respectively, the lowest and highest number sections. The control group used the beginning point most in the lowest number section and the endpoint most in the highest number section. The difference in strategy use between the groups was largest in the highest number section.

On the number line 0–1000 there was a significant multivariate interaction effect between group and strategy use, Wilks’ Lambda = 0.63, *F* (6, 100) = 4.37, *p* = .001, *η*^2^ = .21. The assumption of sphericity was violated for all three number sections, so degrees of freedom for univariate testing have to be corrected by $\hat{\varepsilon }$. Univariate testing showed a large group difference on strategy use on the 0–333 section, *F* (2, 52) = 12.88, $\hat{\varepsilon }$  = .617, *p* = .001, *η*^2^ = .33. Pairwise comparisons showed that the MLD group used the beginning point strategy (*p* = .003) and the midpoint strategy (*p* < .001) significantly more than the endpoint strategy. There was no difference between the use of beginning point and midpoint strategy (*p* = .158). For the control group, all pairwise comparisons on the 0–333 section were significant. The children in this group used the beginning point strategy more than the midpoint (*p* < .007) and endpoint strategy (*p* < .001) and the midpoint strategy more than the endpoint strategy (*p* = .002).

There were no significant univariate interaction effects on the 334–666 section, *F* (2, 52) = 0.31, $\hat{\varepsilon }$  = .598, *p* = .625, *η*^2^ = .01 and the 667–1000 section, *F* (2, 52) = 1.26, $\hat{\varepsilon }$  = .732, *p* = .285, *η*^2^ = .05. Therefore, the strategy use on these number sections was analyzed for both groups together. Pairwise comparisons showed that the midpoint strategy (*p* < .001) was used more than the beginning point (*p* < .001) and the endpoint strategy (*p* < .001) on the 334–666 section. There was no difference in use of the beginning point and endpoint strategy on this number section (*p* = .678). On the 667–1000 section the midpoint (*p* < .001) and the endpoint (*p* < .001) were used more than the beginning point. There was no difference in use of the midpoint and endpoint (*p* = .521).

To summarize, the results on the number line 0–1000 indicated that the MLD group used the beginning point and midpoint equally often in the lowest number section, whereas the control group used the beginning point most often. There were no between-group differences in strategy use on the middle and highest number section.

#### Use of most adaptive strategy per number section

MANOVA’s showed group differences in adaptive strategy use on the number line 0–100, Wilks’ Lambda = 0.75, *F* (3, 24) = 3.04, *p* = .048, *η*^2^ = .28, and on the number line 0–1000, Wilks’ Lambda = 0.49, *F* (3, 24) = 8.34, *p* = .001, *η*^2^ = .51. On the 0–100 number line, there was no difference between the groups in use of the beginning point in the number section 0–33, *F* (1, 26) = 1.47, *p* = .236, *η*^2^ = .05. There was a marginally significant medium to large sized group effect in use of midpoint in the number section 34–66, *F* (1, 26) = 3.79, *p* = .06, *η*^2^ = .13, and a medium effect—although non-significant—in use of endpoint in the number section 67–100, *F* (1, 26) = 2.89, *p* = .101, *η*^2^ = .10. The control group used these reference points more frequently than the MLD group.

On the number line 0–1000 there was a large difference between the groups in use of the beginning point on the number section 0–333, *F* (1, 26) = 16.25, *p* < .001, *η*^2^ = .39. The control group used the beginning point more than the MLD group. There was no difference in the use of the midpoint on the number section 334–666, *F* (1, 26) = 0.08, *p* = .777, *η*^2^ = .00. The difference in use of the endpoint on the number section 667–1000 was not significant, but nevertheless had a medium effect size, *F* (1, 26) = 2.06, *p* = .163, *η*^2^ = .07. The control group made more use of the endpoint than the MLD group.

## Discussion

In this study strategy use on number line estimation in 9–11-year-old children with mathematical learning difficulties and a control group without mathematical learning difficulties was measured using eye tracking. First, it was confirmed that children with MLD have problems with number line estimation on a behavioral level, as reflected by lower linear fit scores and a higher mean percentage absolute error. However, previous research suggests that participants’ estimates on a number line are influenced by their strategy use, more specifically the use of several reference points on the number line (e.g. Newman & Berger, 1984). The current study indeed showed that the estimates of the children with MLD fitted a two-cycle power model less well than the estimates of the children without MLD, suggesting children with MLD make less use of the beginning point, midpoint, and endpoint as a reference to estimate the target number. Our main focus, however, was on the possible differences in strategy use as measured by eye tracking between children with MLD and children without MLD.

### Estimation strategies

Strategy use as measured by eye tracking was first analyzed at task level. The eye tracking data confirmed that children with MLD make less use of reference points, although only the difference in use of the beginning point on the number line 0–1000 was significant. Children with MLD used the beginning point less often as a reference point than children without MLD. Previous research showed that children with MLD make less use of the beginning and endpoint of the line and more use of the midpoint (Van’t Noordende & Kolkman, 2013). A possible explanation for this inconsistency between the current study and previous research is the analysis method. In the study of Van’t Noordende and Kolkman (2013), no threshold for fixations was used and each gaze of the child at one of the reference points was coded as beginning point, midpoint, or endpoint strategy. In the current study, the looking behavior was only coded as beginning, mid- or endpoint strategy when the fixations exceeded the threshold and when there were only fixations on either the beginning, mid-, or endpoints of the number line. This may have led to different percentages in strategy use. There is no clear explanation why the children with MLD made less use of the beginning point than children without MLD, while the groups did not differ in use of the other reference points. The beginning point is the first reference point young children start to use (Ashcraft & Moore, 2012; Friso-van den Bos et al., 2015; Newman & Berger, 1984; Rouder & Geary, 2014; White & Szűcs, 2012), suggesting this is the ‘easiest’ reference point to use. The fact that children with MLD make less use of this ‘easy’ reference point and do not differ on the more difficult midpoint and endpoint suggests that they do not just lag behind in development of strategy use. Future research is recommended to further examine whether children with MLD indeed use different strategies instead of less mature strategies, by comparing children with MLD with younger—ability-matched—children.

### Adaptive strategy use

The second research question in the current study focused on adaptation of strategy use to the number that had to be estimated. An adaptive strategy would be to use reference points close to the target number: the beginning point for smaller numbers, the endpoint for larger numbers, and the midpoint for numbers in between (Newman & Berger, 1984). The current study showed that the MLD-children did not differ largely in their use of the most adaptive strategies compared to the children without MLD. Most of the between-group comparisons on use of the most functional strategy per number section were non-significant. The only significant difference was on the use of the beginning point strategy on the number section 0–333, which was used more by the control group than by the MLD group. However, also non-significant comparisons showed a trend (with medium to large effect sizes) towards less frequent use of adaptive strategies in children with MLD than in children without MLD. This is supported by the significant within-group comparisons on the higher number section on the number line 0–100 and the lower number section on the number line 0–1000. The children with MLD used the midpoint and endpoint strategy and the midpoint and beginning point strategy, respectively, equally often. In contrast, the children without MLD only used one strategy—the most functional one—most often. This suggests children with MLD may have problems choosing the most functional estimation strategy. An alternative explanation for the differences in adaptive strategy use between children with and without MLD would be that children with MLD use reference points farther away from the target number, because their response is also farther away from the target number. Deficits in magnitude representation could have led to estimates that are more deviant from the target number when estimating numbers on the number line (e.g. Geary et al., 2007, 2008). The fixations of children with MLD could be related to these incorrect responses instead of the target number, which leads to use of reference points farther away from the target number. However, the current study showed that the fixations of the children with MLD were also farther away from their own (incorrect) response, making this theory less plausible.

### Conclusion and recommendations for future research

To summarize, this study has shown that tracking eye movements reveals useful information about the number line estimation strategies used by children with MLD. In line with previous research, it shows that number line estimation problems might not only arise from a deficit in magnitude representation, but also from the use of less functional estimation strategies (Schot et al., 2015; Van’t Noordende & Kolkman, 2013; Van Viersen et al., 2013). It should be noted however, that it is difficult to disentangle magnitude representations from strategy use, since understanding of numbers is a prerequisite to use functional strategies. For example, you have to know which reference point is closest to the target number to be able to use the most functional strategy. Moreover, the use of arithmetic procedures is needed to make use of the reference points on the number line (Link et al., 2014). This study has shown that children with MLD make less use of functional estimation strategies, but the underlying cause of these problems is not known yet. A next step would be to further explore the underlying causes of the problems with strategy use in children with MLD and possible methods to train functional strategy use.

A possible limitation of our data analysis technique is that dividing the number line in three equal sections of one-third of the number line each is somewhat arbitrary. For numbers near the border of the section, fixations very close to the number might still fall in the adjacent section. However, alternatives, for example a bandwidth around the number do not fit with the observation that the majority of fixations is centered on the beginning, mid- and endpoint of the number line (Van’t Noordende & Kolkman, 2013). In addition, a possible limitation of the current study is that the target number was displayed in the middle underneath the number line and might have been used as an external reference point. Although it seems unlikely that this would cause a difference between the two groups since the task was the same for the children with and without MLD, future research could vary the position of the target number or present it verbally.

Another suggestion for future research would be to look at individual differences. The data in the current study were analyzed at group level, although the individual fixation plots (see Online Resource 2 and Fig. 1) show there is also variation within groups. Schot et al. (2015) suggested that individual differences in children with MLD might be caused by the severity of the MLD or by different subtypes of MLD. In the current study, no information about the subtypes of MLD was available. It would be interesting to explore possible differences in estimation strategy use between children with different subtypes of MLD in a larger sample.

## Electronic supplementary material

Below is the link to the electronic supplementary material.

Supplementary material 1 (PDF 514 kb)

Supplementary material 2 (PDF 366 kb)

## Footnotes

1 The sample in this study was the same as in a previously published article by the first author in a Dutch journal (Van’t Noordende & Kolkman, 2013). However, a newly developed, more standardized, analysis method was used in the current article to analyze the data. Moreover, the data were analyzed in the current study on both task and item levels instead of only at task level.

2 Note that, as the number line is always divided into three equal segments, only numbers in the first one-third and in the last one-third of the number line can have fixations that are two segments away from the correct segment. This does not pose a problem for the data analysis as all children estimated the same numbers.
