# Supplementary material for: Number line estimation strategies in children with mathematical learning difficulties measured by eye tracking
Source: Psychol Res. 2015 Dec 26;80:368–78. doi: 10.1007/s00426-015-0736-z (PMC4826415; doi:10.1007/s00426-015-0736-z)
Supplement: Supplementary file 1 — Supplementary material 1 (PDF 514 kb) [file 426_2015_736_MOESM1_ESM.pdf]

Online Resource 1

Article: Number line estimation strategies in children with mathematical learning difficulties measured by eye tracking

Journal: Psychological Research

Authors:

Jaccoline E. van ’t Noordende (J.E.vantNoordende@uu.nl)

Anne H. van Hoogmoed

Willemijn D. Schot

Evelyn H. Kroesbergen

Department of Special Education: Cognitive and Motor Disabilities

Utrecht University, The Netherlands

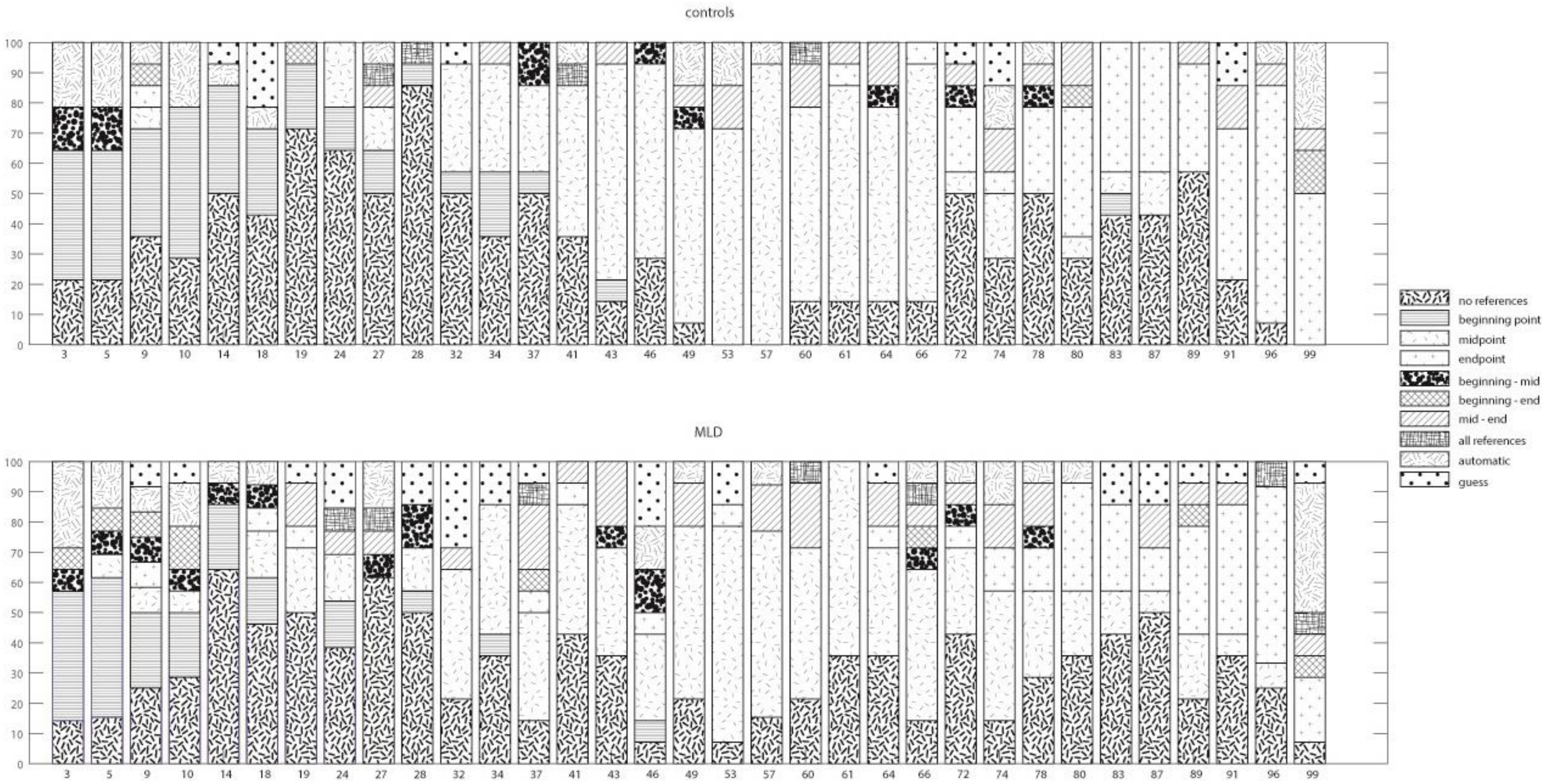

Fig. 1 Percentage strategy use per trial (target number) on the number line 0-100.

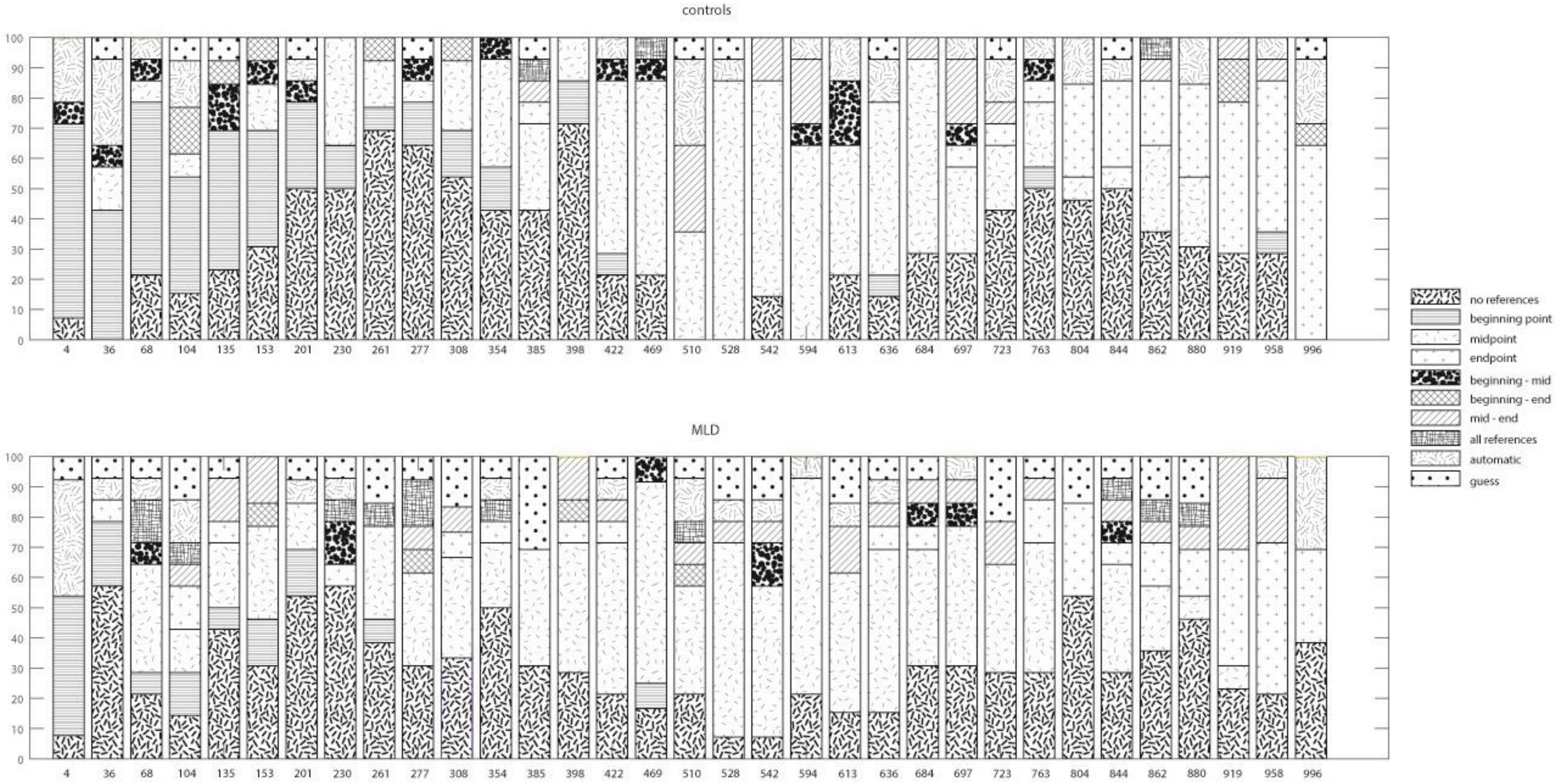

Fig. 2 Percentage strategy use per trial (target number) on the number line 0-1000.
