# Supplementary material for: Number line estimation strategies in children with mathematical learning difficulties measured by eye tracking
Source: Psychol Res. 2015 Dec 26;80:368–78. doi: 10.1007/s00426-015-0736-z (PMC4826415; doi:10.1007/s00426-015-0736-z)
Supplement: Supplementary file 2 — Supplementary material 2 (PDF 366 kb) [file 426_2015_736_MOESM2_ESM.pdf]

Online Resource 2

Article: Number line estimation strategies in children with mathematical learning difficulties measured by eye tracking

Journal: Psychological Research

Authors:

Jaccoline E. van ’t Noordende (J.E.vantNoordende@uu.nl)

Anne H. van Hoogmoed

Willemijn D. Schot

Evelyn H. Kroesbergen

Department of Special Education: Cognitive and Motor Disabilities

Utrecht University, The Netherlands

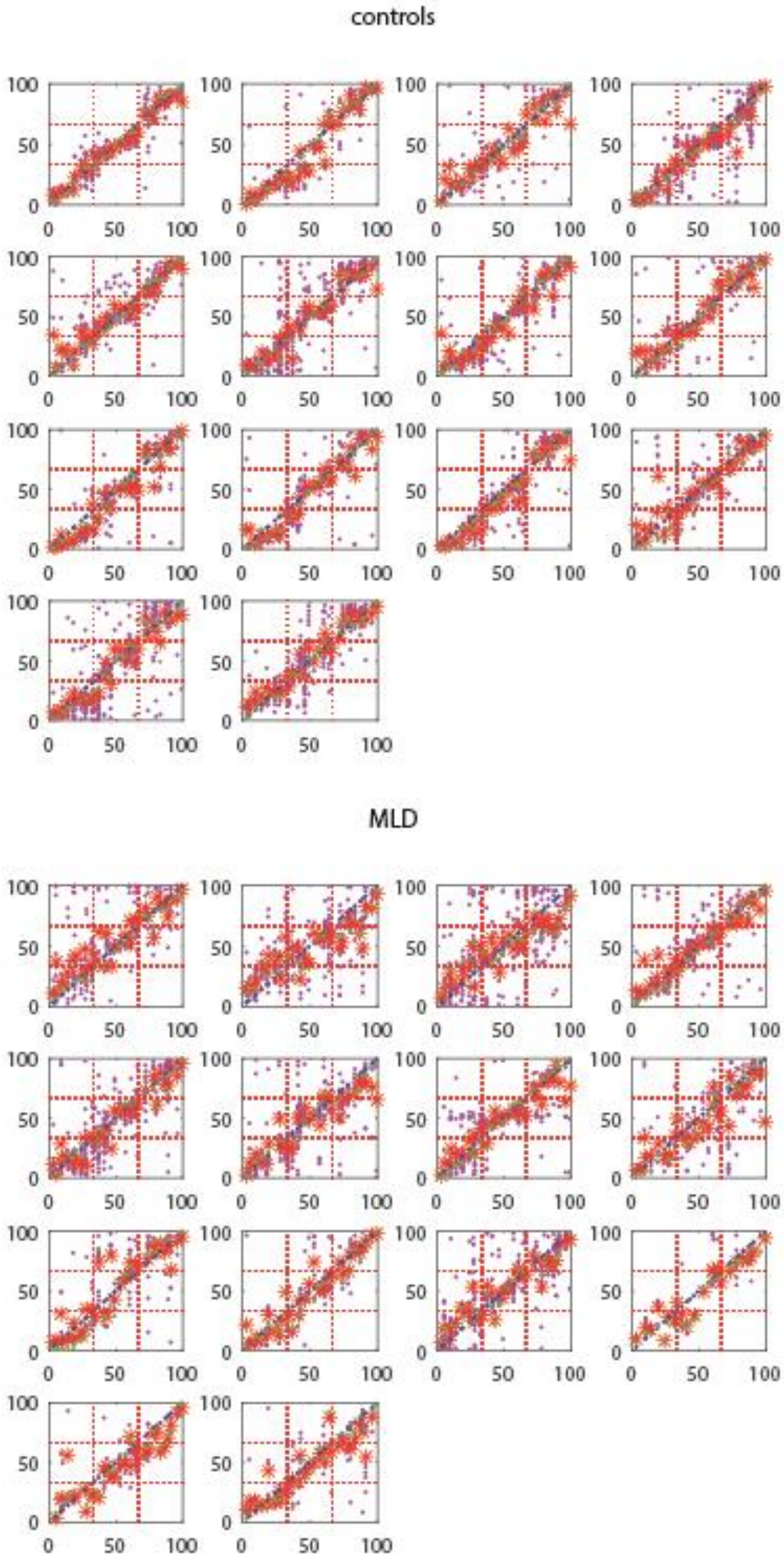

Fig. 1 Fixations and responses in relation to the target number on the number line 0-100. Each panel represents the data of one participant. Blue dots represent the target number, green dots represent the response, pink dots represent all fixations (separately) and red stars represent mean fixation per trial.

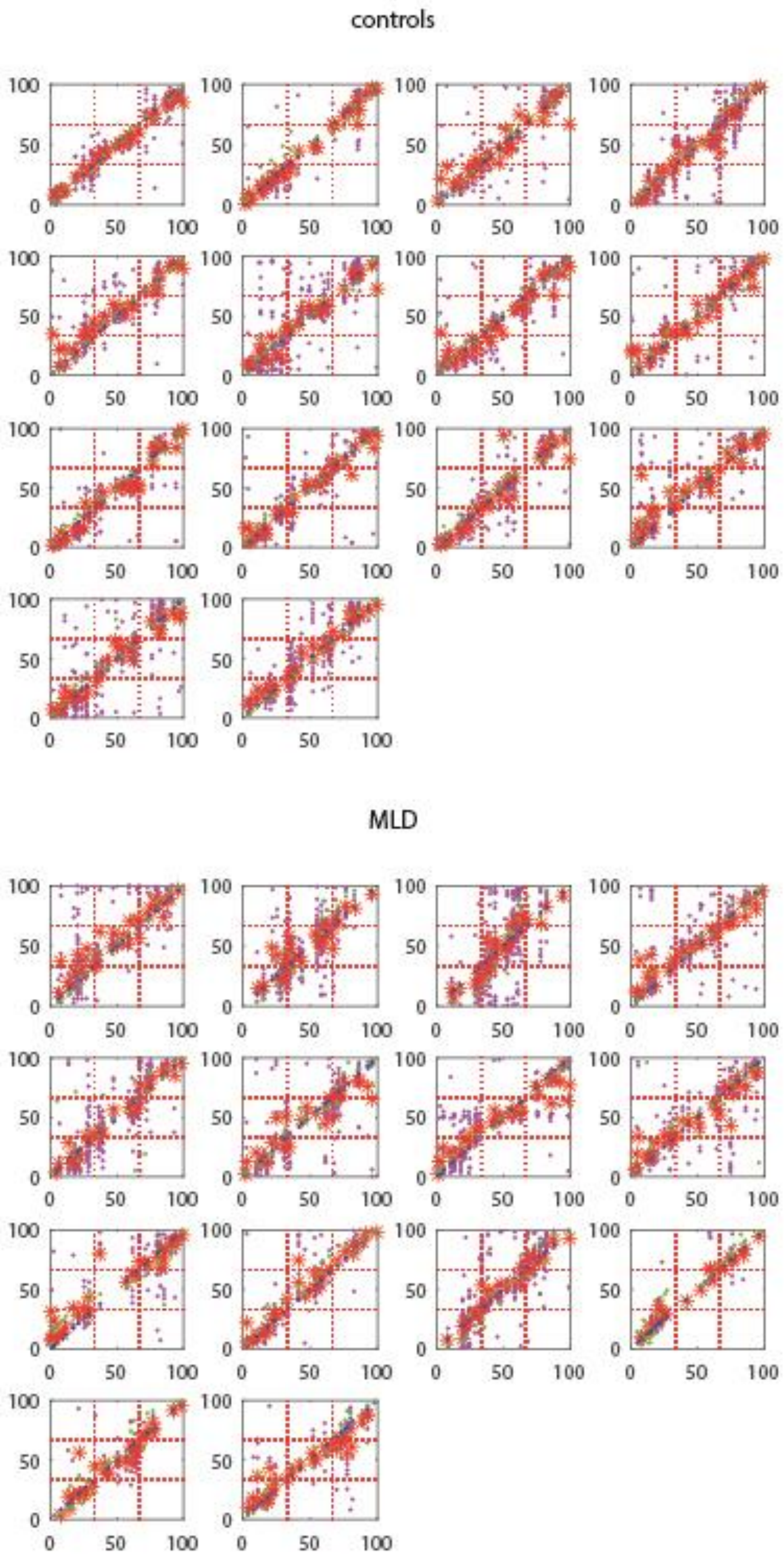

Fig. 2 Fixations and responses in relation to the response on the number line 0-100. Each panel represents the data of one participant. Blue dots represent the response, green dots represent the target number, pink dots represent all fixations (separately) and red stars represent mean fixation per trial.

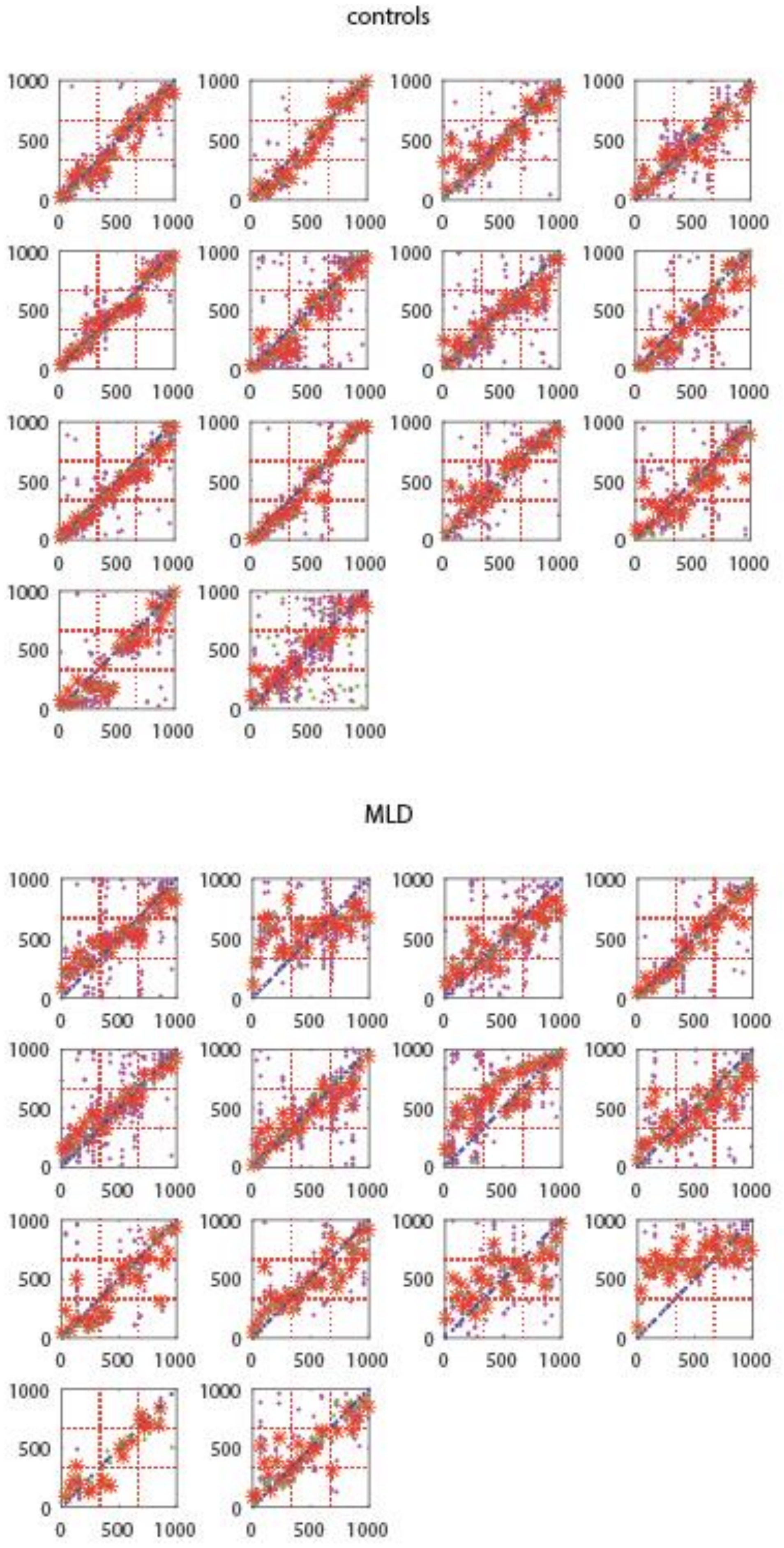

Fig. 3 Fixations and responses in relation to the target number on the number line 0-1000. Each panel represents the data of one participant. Blue dots represent the target number, green dots represent the response, pink dots represent all fixations (separately) and red stars represent mean fixation per trial.

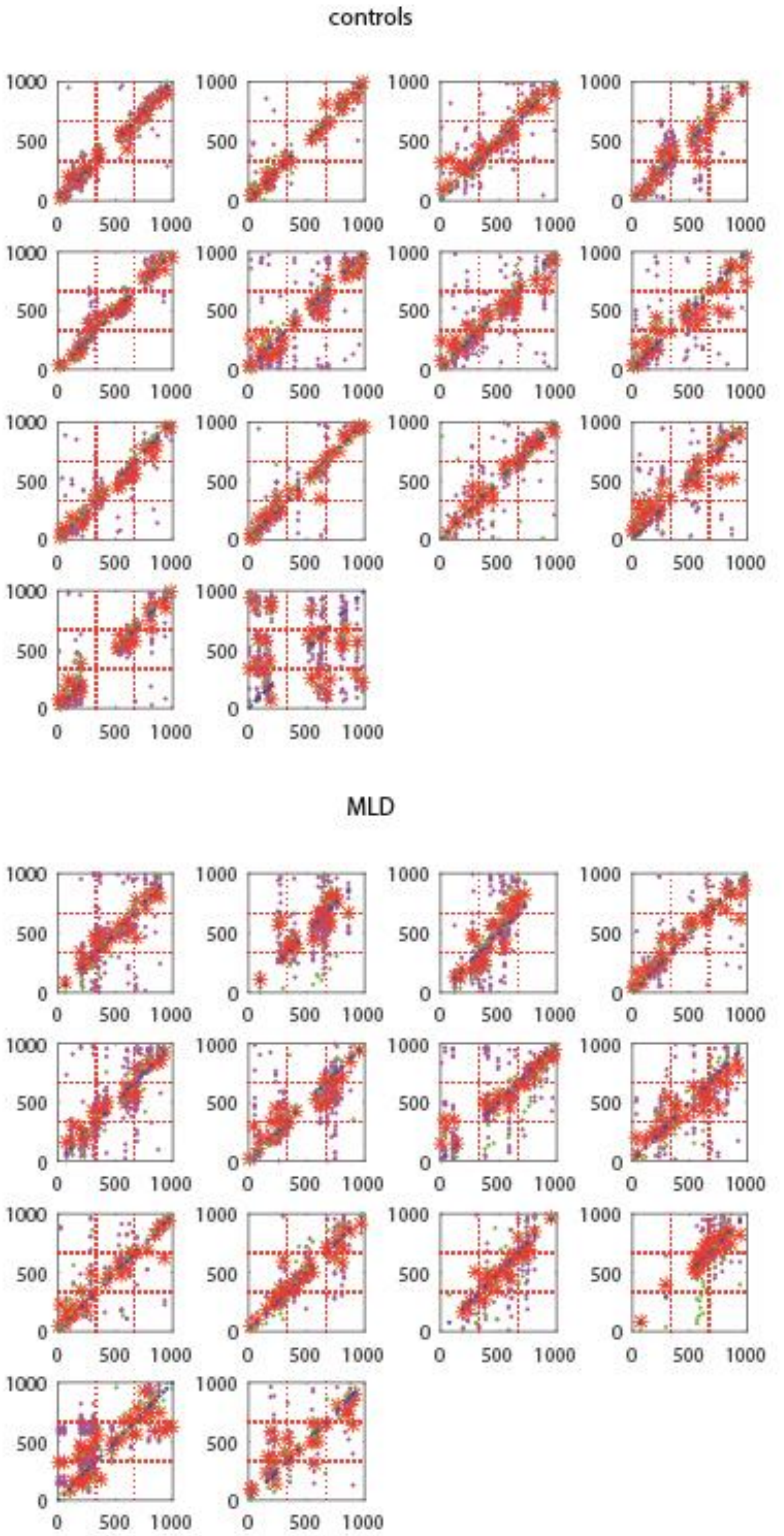

Fig. 4 Fixations and responses in relation to the response on the number line 0-1000. Each panel represents the data of one participant. Blue dots represent the response, green dots represent the target number, pink dots represent all fixations (separately) and red stars represent mean fixation per trial.
